# Improvements on Clinical Status of Adolescents With Anorexia Nervosa in Inpatient and Day Hospital Treatment: A Retrospective Pilot Study

**DOI:** 10.3389/fpsyt.2021.653482

**Published:** 2021-05-28

**Authors:** Valeria Zanna, Giulia Cinelli, Michela Criscuolo, Anna Maria Caramadre, Maria Chiara Castiglioni, Ilenia Chianello, Maria Rosaria Marchili, Chiara Casamento Tumeo, Stefano Guolo, Alberto Eugenio Tozzi, Stefano Vicari

**Affiliations:** ^1^Anorexia Nervosa and Eating Disorder Unit, Child Neuropsychiatry, Department of Neuroscience, Bambino Gesù Children's Hospital, Istituto di ricovero e cura a carattere scientifico (IRCCS), Rome, Italy; ^2^Predictive and Preventive Medicine Research Unit, Bambino Gesù Children's Hospital, Istituto di ricovero e cura a carattere scientifico (IRCCS), Rome, Italy; ^3^General Pediatrics Unit, Department of Emergency, Acceptance and General Pediatrics, Bambino Gesù Children's Hospital, Istituto di ricovero e cura a carattere scientifico (IRCCS), Rome, Italy; ^4^Sanitary Direction, Bambino Gesù Children's Hospital, Istituto di ricovero e cura a carattere scientifico (IRCCS), Rome, Italy; ^5^Child and Adolescent Psychiatric Unit, Department of Neuroscience, Bambino Gesù Children's Hospital, Istituto di ricovero e cura a carattere scientifico (IRCCS), Rome, Italy

**Keywords:** daily treatment, inpatient care, hospital care, partial hospitalization, anorexia nervosa, adolescence

## Abstract

**Introduction:** Medical and psychiatric complications and treatment compliance are important considerations in determining the treatment program for patients with severe anorexia nervosa (AN). Clinical practice guidelines agree that an outpatient program is the first choice for the treatment of most eating disorders, but vary in supporting these programs for AN. However, inpatient care is known to be costly and the risk of relapse and readmission is high. This pilot study aimed to describe the first data on an Italian partial hospitalization care program for AN adolescents [high-level care treatment (HLCT)], evaluating its impact on patients' clinical status, average hospitalization time, and the hospital costs compared to inpatient treatment (IP).

**Methods:** For this retrospective pilot study, we have selected a group of 34 females with AN aged 11–18 years, divided between those who followed inpatient treatment and those who received HLCT treatment; they were matched for age and severity. We investigated the differences in treatment and outcomes between the two groups in terms of heart rate, length of treatment, weight gain, psychological characteristics, and hospital costs. Statistics for non-parametric distributions were used to compare the two groups.

**Results:** No differences between the two groups were found at admission. At discharge, patients in the HLCT group presented a lower number of in-hospital treatment days, a higher increase of weight, and a significant improvement in outcomes compared to the inpatient group. No significant differences were found in heart rate and hospital costs.

**Conclusions:** This study represents a first comparison between inpatient care and the HLCT treatment program, which suggests that day hospital treatment could represent a meeting point between inpatient and outpatient treatment, combining the merits of both forms of treatment. Further studies are needed in order to better investigate the different treatment programs for severe AN in adolescence.

## Introduction

According to the most recent data published by the Italian Ministry of Health, anorexia nervosa (AN) is the most common problem among young people, with an estimated incidence of at least 8 new cases per 100,000 women in a year, and it is constantly growing in the male population (1). The International guidelines on clinical practice for eating disorders in childhood and adolescence (2–4) point out that the integrated multidisciplinary outpatient treatment model is the most suitable intervention for AN and guarantees adequate care response in 70% of cases. Outpatient treatment provides care in a non-restrictive setting: it preserves the patient's sense of autonomy, allowing for and improving their ability to maintain normal social and work activities, and is perceived as more syntonic, favoring patient compliance (5). Moreover, studies have shown that it is more effective and efficient in terms of time and cost of therapy compared to inpatient treatment (IP).

However, in cases of moderate to severe AN, or when outpatient treatment is not effective, impatient care (IP) could be the treatment of choice. Severe AN in adolescence is defined not only by clinical and laboratory data (BMI, hearth rate, blood pressure, etc.) but also by the rate of weight loss and the caloric intake (6).

Previous systematic reviews have compared different therapeutic treatment programs: outpatient, IP, and day patient (DP) for adolescent AN. They found no differences in outcomes as measured by changes in weight, eating disorder pathology, or lengths of treatment (7). Moreover, recent studies have underlined that IP presents substantial financial costs as well as leads to higher relapse and readmission rates than the other forms of treatment (8). DP treatment is considered to be the central treatment for subintensive psychiatric patients and for performing medical interventions or as an alternative to the IP setting (6). A recent study by Herpertz-Dahlmann et al. (9) have compared DP treatment following a short stay for inpatient care to continued IP. Their results have found the same efficacy for DP care compared to IP care for weight restoration and maintenance during the first year after admission, with less costs than a IP program.

Both care programs, inpatient and day hospital treatments, are usually multidisciplinary with a combination of health specialists, intensive medical and psychotherapy assistance, nutritional counseling, and supervised meals (10). Usually, the main difference is that in DP there is no overnight stay (9).

In IP and DP programs, the prolonged periods of care allow the team to directly control meals, quickly respond to psychiatric or physical emergencies, and provide psychological support, increasing adherence to the prescribed meal plan (10, 11). Moreover, greater frequency of therapy sessions leads to more rapidly acquired psychological knowledge and skills (4).

Based on the evidence available to date and in order to offer the most appropriate care program for patients' needs, the Bambino Gesù Children Hospital Unit of Anorexia Nervosa and Eating Disorders has implemented a day hospital care program named high-level care treatment (HLCT). This care model was created to address the riskiest situations without the use of hospital IP. Admission criteria use the same parameters as those for IP admissions, which exclude the most medically unstable patients. The HLCT program utilizes a treatment plan halfway between the high-frequency clinical monitoring and assistance with meals, characteristic of the IP program, and the possibility of maintaining social and family spaces, a characteristic of the 1-day-a-week DP program, multifocal integrated treatment (MIT), already in place in the hospital (12). The HLCT therapeutic program provides assistance from a multidisciplinary team consisting of a psychiatrist, psychotherapists, and a nutritionist. The treatment is organized 3 days a week, which includes nutritional assistance at lunch and one snack time as well as psychiatric and individual nutritional checks at every session. In addition, there are separate therapeutic groups for parents and patients, and psychoeducational multifamily groups, which are scheduled weekly. As in the IP program, where enteral nutrition is often activated in cases of medical needs, bolus administration can also be started in the HLCT program if the patient's clinical conditions require it. The hypothesis underlying this approach is that the skills learned by patients and parents during treatment can be more easily transferred into everyday life and that patients themselves may be able to maintain contact with their social networks, thus supporting social competence and experiencing treatment in a less restrictive way than in the IP approach. However, the actual effectiveness of partial hospital treatments, such as HLCT, compared with the IP programs and the differences in hospital costs in Italy were as yet unknown. This study aims to describe the first available data on an Italian partial hospitalization program—the HLCT treatment—in order to evaluate the impact on patients' clinical status (in terms of weight recovery and mental state), average hospitalization days, and hospital costs compared to the IP program for adolescents with AN.

Results will be useful to build a structured clinical trial, modeled on that previously done by Herpertz-Dahlmann et al. (9), which promotes a more in-depth investigation of the treatment indications for patients with severe AN in different types of treatment programs.

## Materials and Methods

### Subjects and Study Design

The pilot study has an observational retrospective design, so there was no opportunity to work on the composition of the sample. To create two comparable groups, we have selected patients diagnosed with AN admitted from December 2019 to September 2020 to the Bambino Gesù Children Hospital at the Pediatric Unit for an IP treatment program. Inclusion criteria were as follows: male and female, all ages, and primary diagnosis of AN based on DSM-5 criteria. Patients with intellectual disabilities, pervasive developmental disorders, other neurological conditions, and a non-AN primary diagnosis were excluded. After a selection of patients from the IP group, we selected a second group of patients admitted at the Anorexia Nervosa and Eating Disorder Unit using the HLCT program, matched for age, BMI, and clinical status.

All the patients included in the present study underwent an evaluation for the diagnosis (T0) consisting of nutritional assessment and psychological and psychiatric assessment (12). Family history of anxiety, depression, or eating disorder was evaluated up to second-degree relatives.

The diagnostic assessment was made at the moment of referral by a trained psychiatrist (V.Z.), who first made the diagnosis through a routine clinical interview and then used the Italian version of the Schedule for Affective Disorders and Schizophrenia for School-Age Children/Present and Lifetime Version (K-SADS-PL DSM-5) (13) to confirm the diagnosis as well as other psychiatric comorbidities. The following clinical parameters were collected at the time of admission (T0) and at the time of discharge (T1): weight, height, percentiles of body mass index (pBMI) and heart rate (HR). pBMI shows how the child's weight compares to that of other children of the same age and sex and was determined using the 2,000 Center for Disease Control and Prevention growth charts (CDCP). A pBMI lower than the first percentile was defined in the analysis as 0.5, by convention. Outcomes at T1 were also evaluated with a specific assessment scale [Morgan–Russel Outcome Assessment Scale (MROAS)] (14, 15). Primary amenorrhea was defined as the absence of spontaneous menstruation by 15 years of age with normal development of secondary sexual characteristics (16).

The study was reviewed and approved by the Ethical Committees of the Bambino Gesù Children Hospital (2264_OPBG_2020). Written informed consent to participate in this study was provided by the participants' legal guardian/next of kin.

### Psychological Measures

During the clinical assessment at T0, each patient received a package containing the psychometric battery of self-administered questionnaires and was asked to complete them. Later, psychologists scored all of the questionnaires. Emotional and behavioral characteristics and psychopathological dimensions were assessed with the Multidimensional Anxiety Scale for Children 2 (MASC 2) (17, 18), the Children Depression Inventory 2 (CDI 2) (19, 20), and the Youth Self-Report (YSR) (21, 22). Eating disorder psychopathology and the possible presence of dysmorphophobia were investigated using the Eating Disorder Inventory-3 (EDI-3) (23, 24) and the Body Uneasiness Test (BUT) (25), respectively. Family functioning was assessed with the Family Assessment Device (FAD) (26). Finally, the patients' clinical course was evaluated at T1 using the MROAS (14) in the version modified by Jeammet et al. (15). Considering the low sample size and the risk of imprecise estimates, we decided to not calculate the internal consistency for the self-report measures but to report the reliability coefficients of the validation studies.

#### Multidimensional Anxiety Scale for Children 2

The Multidimensional Anxiety Scale for Children 2 (MASC 2) is a questionnaire for the evaluation of the main dimensions of anxiety in children and adolescents from 8 to 19 years of age. The self-report form contains 50 items, which measure Separation/Fears, Generalized Anxiety (GAD) Index, Obsessions/Compulsions, Harm Avoidance, Social Anxiety (Humiliation/Rejection and Performance Fears), and Physical Symptoms (Panic and Tense/Restless). The Italian version of MASC 2 has shown excellent validity, like the original version, a good internal consistency, and test–retest reliability (18).

#### Children Depression Inventory 2

The Children Depression Inventory 2 (CDI 2) is a self-report questionnaire for the evaluation of the depressive symptoms of children and adolescents from 7 to 17 years of age. It is made up of sets of items, each containing three options that reflect the severity of the symptom, from 0 (absent) to 2 (defined, marked). From the self-report form, clinicians get a Total Score as well as scores on two different scales: Emotional Problems and Functional Problems. In addition, it provides scores for four further subscales, called Negative Mood/Physical Symptoms, Negative Self-Esteem, Ineffectiveness, and Interpersonal Problems. The set of statistical surveys highlighted the quality of the test items, their reliability, and the validity of the Italian version (19).

#### Youth Self-Report

To assess the adolescents' view of their behavior and socioemotional functioning, the Italian version of the YSR was used. This questionnaire has to be completed by an 11- to 18-year-old adolescent and contained 112 problem items, covering behavioral, emotional, and social problems that occurred during the past 6 months. The YSR can be scored on syndrome scales: Anxious/Depressed, Withdrawn/Depressed, Somatic Complaints, Social Problems, Thought Problems, Attention Problems, Aggressive Behavior, and Rule-Breaking Behavior. The Internalizing scale can be derived from the first three syndrome scales, and the Externalizing scale can be derived from the last two. This measure, in its validated Italian version, has demonstrated very good day test–retest reliability, cross-informant agreement, and success in discriminating between referred and no referred adolescents (22).

#### Eating Disorder Inventory-3

The Eating Disorder Inventory-3 (EDI-3) is a self-report instrument measuring psychological traits or constructs shown to be clinically relevant in individuals with eating disorders. This measure consisted of 91 items organized into 12 primary scales, three eating disorder-specific scales (Drive for Thinness—DT; Bulimia—B; Body Dissatisfaction—BD), and 9 general psychological scales (Low Self-Esteem—LSE; Personal Alienation—PA; Interpersonal Insecurity—II; Interpersonal Alienation—IA; Interoceptive Deficits—ID; Emotional Dysregulation—ED; Perfectionism—P; Asceticism—A; Maturity Fears—MF) that are highly relevant to, but not specific to, eating disorders. The reliability coefficients of the scales range from 0.83 and 0.90, and test–retest reliability coefficients for the various composite scales are between 0.84 and 0.87. The Italian version of EDI-3 (24) has demonstrated adequate indices of validity and reliability with reliability indices ranging from 0.90 and 0.97 calculated on the total sample of the Italian validation study.

#### Body Uneasiness Test

The Body Uneasiness Test was used for the clinical assessment of body uneasiness. The BUT-A consists of four subscales and a global severity index (GSI) that have been demonstrated to have good internal consistency and reliability: Weight Phobia (WP—fear of being or becoming fat), Body Image Concerns (BIC—worries related to physical appearance), Avoidance (A—body image-related avoidance behavior), Compulsive Self-Monitoring (CSM—compulsive checking of physical appearance), and Depersonalization (D—detachment and estrangement feelings toward the body). The Italian version of the instrument shows good reliability coefficients and a factorial structure congruent with the operative definition of the construct (25).

#### Family Assessment Device

The FAD is a 60-item self-report questionnaire for assessing participants' views of their family functioning. The FAD was administered to both parents and patients, but only the latter's versions were used for the present study. Scoring produced ratings on seven aspects of family functioning: problem-solving, communication, roles, affective responsiveness, affective involvement, behavior control, and general functioning. Lower scores indicate healthier functioning than higher scores. The Italian version of FAD has been shown to have good reliability coefficients (27).

#### Morgan–Russel Outcome Assessment Scale

The Morgan–Russell Average Outcome Score MRAOS is a scale for the biopsychosocial assessment of the treatment outcomes, compiled by the clinician at the end of treatment, based on information received from the patient or observed during treatment. The MROAS is derived from a guided interview assessing core clinical features of AN. The clinician rated each item with a score from 1 (satisfactory) to 6 (very unsatisfactory). Following this procedure, patients are divided into three groups, depending on their scores: good when at least eight items have been rated 1 or 2; intermediate, if four to seven items have been rated 1 or 2; poor if three items or less have a score of 1 or 2 (14, 15).

### Cost Assessment

The cost assessment was performed using the Health Care Financing Administration-Diagnosis Related Group (HCFA-DRG) system, version 24 (28). In the Italian healthcare system, hospitalizations are reimbursed according to a system, which has a national reference and is adjusted on a regional basis. For this reason, in this study, the Lazio Region Tariff Nomenclature DRG of outpatient services was used. A specific DRG is applied to the discharge diagnosis of each patient, and corresponds to a specific cost. In case of IP, there is a fixed cost if the length of stay is below a threshold. In case of AN, the threshold is 41 days. If a patient has a length of stay over the threshold, the hospitalization costs are calculated, adding a daily rate for days exceeding the threshold. On the contrary, for the HLCT, costs are calculated on a daily basis.

### Statistical Analysis

For the analysis, patients were divided into two groups according to the admission type (IP group and HLCT group) in order to compare their individual characteristics. The Shapiro–Wilk test was performed in order to evaluate variable distribution. The variables had both normal and skewed distribution, but due to the small sample size, a non-parametric analysis was performed. Data are represented as number and percentage in parentheses (%) for categorical variables, or median and interquartile range in square brackets [IQR] for continuous variables. The Mann–Whitney *U* test was performed in order to compare continuous and ordinal variables between the two groups, while the Chi-Square test was used for categorical ones. The Wilcoxon test was performed to investigate intragroup changes between T0 and T1. The effect size for non-parametric tests was calculated. The *r* proposed by Cohen was used for the Mann–Whitney *U* and the Wilcoxon tests, with small, medium, and large effects for *r* < 0.3, 0.3 ≤ *r* < 0.5, and *r* ≥ 0.5, respectively (29). Cramer's *V* was used for the Chi-square test (30). Statistical analysis was performed through IBM SPSS Statistics V21.0.

## Results

### Subjects

Sixty-four subjects were selected for this study: 23 were excluded for missing data, five were excluded for the presence of binge/purging behaviors, one for comorbidity with other organic diseases, and one for previous history of avoidant/restrictive food intake disorder. Finally, 34 patients were included in the analysis, 17 for each group. All of the patients hospitalized in the IP program (100%) accessed our hospital through the emergency room (ER) (Cramer's *V* = 0.692) while only six (35.3%) of those that were then sent to the HLCT program did. No difference was found between the two groups at T0 with respect to clinical and psychological variables, or other parameters such as length of illness, rate of weight loss, time of weight loss, and prior treatments (Table 1). Differences were found in the number of hospital treatment days, which were lower in the HLCT group (Mann–Whitney *U* = 54.5000, *p* = 0.001, *r* = 0.533), and in the use of fluid therapy and enteral nutrition, which were more frequent in the IP group (*p* < 0.001, Cramer's *V* = 0.943; *p* < 0.001, Cramer's *V* = 0.600), with a large effect size.

**Table 1:** Patients' characteristics in IP and HLCT groups.

	**IP (*n* = 17)**	**HLCT (*n* = 17)**	**Effect size (*r* or Cramer's *V*)**
Sex (Female)	15 (88.2)	16 (94.1)	0.104
Age (y)	15.28 [14.11–15.61]	14.35 [13.77–16.25]	0.027
Amenorrhea			0.349
Absent	1 (5.9)	1 (5.9)	
Pre-puberty	1 (5.9)	4 (23.5)	
Primary	2 (11.8)	0 (0.0)	
Secondary	11 (64.7)	11 (64.7)	
Length of illness (m)	9.00 [6.00–18.00]	8.00 [6.00–12.00]	0.080
Total weight loss (kg)	15.00 [8.00–20.00]	14.00 [10.00–20.00]	0.012
Time of weight loss (m)	8.00 [5.00–12.00]	6.00 [3.00–8.00]	0.252
Previous treatments			0.376
None	8 (47.1)	11 (64.7)	
Ordinary Hospitalization	0 (0.00)	2 (11.8)	
Other treatment	8 (47.1)	4 (23.5)	
Both	1 (5.9)	0 (0.00)	
Comorbidity			0.174
Depressive disorders	1 (5.9)	1 (5.9)	
Specific Learning Disabilities	0 (0.0)	1 (5.9)	
Family (United)	15 (88.2)	12 (70.6)	0.218
Family history			
Anxiety disorders	1 (5.9)	3 (17.7)	0.183
Depressive disorders	1 (5.9)	5 (29.4)	0.309
Eating disorders	2 (11.8)	1 (5.9)	0.104
Other	2 (11.8)	0 (0.0)	0.250
Length of treatment (d)	30.00 [27.00–36.00]	32.00 [19.00–51.00]	0.094
Number of in-hospital treatment days (d)	30.00 [27.00–36.00]	19.00 [13.00–27.00]^**^	0.533
Drug therapy (yes)	16 (94.12)	14 (82.35)	0.183
Drug type			0.381
SSRI	0 (0.0)	1 (5.9)^***^	
Atypical antipsychotics	4 (23.5)	6 (35.3)	
SSRI + Atypical antipsychotics	10 (58.8)	7 (41.2)	
Other	2 (11.8)	0 (0.0)	
Fluid therapy	17 (100.0)	1 (5.9)	0.943
Enteral nutrition	9 (52.9)	0 (0.0)^***^	0.600
Supplemental nutrition	14 (82.4)	13 (76.5)	0.073

*Data are presented as number and percentage in parentheses (%) for categorical variables, or median and interquartile range in square brackets [IQR] for continuous variables. The “length of treatment” refers to the total number of days between the admission and the discharge. Conversely, the “number of in-hospital treatment days” refers to the actual number of days patients accessed the hospital and the treatment. d, days; kg, kilograms; m, months; IP, inpatient care; HLCT, high-level care treatment; SSRI, selective serotonin reuptake inhibitor; y, years. Mann–Whitney and Chi-square tests were used to compare continuous and categorical variables, respectively. Statistical significance for p < 0.05*.

** *p < 0.01*,

*** *p < 0.001. Effect size was expressed as r or Cramer's V for continuous and categorical variables, respectively. Small, medium, and large effects for r < 0.3, 0.3 ≤ r < 0.5, and r ≥ 0.5, respectively*.

### Anthropometrics

No difference was found in the clinical parameters between the two groups at T0 and T1 (Table 2). Both groups showed an increase in weight at T1 (Delta Weight in kg: IP = 1.40 [0.20–2.20], *p* = 0.010, *r* = 0.443; HLCT = 2.40 [1.70–3.90], *p* < 0.001, *r* = 0.603), with higher effect size values in the HLCT group compared to the IP group. Moreover, an increase in pBMI with a large effect size was detected only in the HLCT group (*p* = 0.001, *r* = 0.546).

**Table 2:** Clinical parameters at T0 and T1 in IP and HLCT patients.

	**IP**		**HLCT**		**Effect size (** ***r*** **)**		
	**T0**	**T1**	**T0**	**T1**	**IP vs. HLCT at T0^a^**	**IP T0 vs. T1^b^**	**HLCT T0 vs. T1^b^**
Weight (kg)	36.00 [32.50–39.50]	37.90 [35.10–42.70]^*^	39.30 [36.20–41.00]	41.60 [38.80–43.50]^***^	0.162	0.443	0.603
Height (cm)	158.00 [150.00–162.00]	158.00 [150.00–162.00]	160.00 [151.00–162.50]	160.00 [152.00–163.00]	–	–	–
pBMI	0.50 [0.50–5.00]	3.00 [0.50–9.00]	2.00 [0.50–6.00]	10.00 [2.00–28.00]^**^	0.116	0.242	0.546
HR (pbm)	62.00 [50.00–74.00]	65.00 [62.00–70.00]	62.00 [60.00–74.00]	72.00 [68.00–83.00]	0.124	0.062	0.273

*Data are presented as median and interquartile range in square brackets [IQR] for continuous variables. Bpm, beats per minute; IP, inpatient care; HLCT, high-level care treatment. The Mann–Whitney test was used to compare continuous variables between the groups at T0. No difference was found. The Wilcoxon test was performed to investigate intragroup changes between T0 and T1. Statistical significance for p < 0.05*.

* *p < 0.05*,

** *p < 0.01*,

*** *p < 0.001. Effect size (r) was calculated for the Mann–Whitney test^a^ and the Wilcoxon test^b^. Small, medium, and large effects for r < 0.3, 0.3 ≤ r < 0.5 and r ≥ 0.5, respectively*.

### Psychological Measures

Considering the psychological evaluations administered at T0, differences were found between groups for the CDI negative self-esteem score, which was higher in the IP group (Mann–Whitney *U* = 66.000, *p* = 0.011, *r* = 0.441), and for the FAD problem solving score, which, in contrast, was higher in the HLCT group (Mann–Whitney *U* = 143.000, *p* = 0.029, *r* = 0.421). Both tests showed a medium effect size (Table 3).

**Table 3:** Patients' psychopathological characteristics in IP and HLCT groups at T0.

	**IP**	**HLCT**	**Effect size (*r*)**
Internalizing problems (YSR)	64.00 [57.00–71.00]	60.00 [52.50–65.50]	0.242
Externalizing problems (YSR)	51.00 [42.00–56.00]	49.50 [46.50–55.50]	0.025
Total problems (YSR)	61.00 [54.00–63.00]	54.50 [47.50–60.00]	0.223
Affective problems (YSR)	66.00 [61.00–70.00]	62.00 [56.00–78.00]	0.207
Anxiety problems (YSR)	59.00 [55.00–63.00]	57.50 [52.00–61.00]	0.111
Somatic problems (YSR)	56.00 [51.00–56.00]	53.00 [52.00–60.00]	0.019
ADHD problems (YSR)	51.00 [50.00–54.00	51.00 [50.00–52.00]	0.109
Oppositional defiant problem (YSR)	52.00 [51.00–65.00]	52.00 [50.5–60.00]	0.083
Conduct problems (YSR)	50.00 [50.00–51.00]	50.00 [50.00–51.00]	0.059
Obsessive-compulsive problems (YSR)	63.00 [63.00–70.00]	56.50 [52.00–64.00]	0.334
Post-traumatic stress problems (YSR)	65.00 [52.00–70.00]	59.00 [52.50–63.00]	0.204
Positive qualities (YSR)	44.00 [38.00–48.00]	46.00 [40.00–54.50]	0.170
Total score (CDI 2)	62.00 [54.00–75.00]	54.50 [42.50–59.00]	0.336
Emotional problems (CDI 2)	69.00 [57.00–74.00]	56.00 [51.50–63.50]	0.333
Negative mood/physical symptoms (CDI 2)	65.00 [54.00–72.00]	58.00 [50.00–68.00]	0.154
Negative self-esteem (CDI 2)	67.00 [54.00–74.00]	50.00 [45.00–56.50]^*^	0.441
Interpersonal problems (CDI 2)	67.50 [52.50–76.50]	57.50 [53.00–61.00]	0.230
Ineffectiveness (CDI 2)	65.00 [42.00–68.00]	50.00 [44.50–57.50]	0.220
Interpersonal problems (CDI 2)	59.00 [47.00–66.00]	52.00 [41.00–59.00]	0.263
Total score (MASC 2)	58.00 [46.00–66.00]	55.00 [50.00–67.00]	0.015
Separation anxiety (MASC 2)	50.00 [42.00–60.00]	57.00 [40.00–60.00]	0.018
GAD index (MASC 2)	55.00 [47.00–63.00]	61.00 [48.00–65.00]	0.160
Social anxiety (MASC 2)	51.00 [44.00–59.00]	52.00 [45.00–59.00]	0.068
Humiliation/rejection (MASC 2)	44.00 [41.00–59.00]	53.00 [43.00–58.00]	0.030
Performance fears (MASC 2)	57.00 [46.00–64.00]	56.00 [46.00–60.00]	0.065
Obsessions & compulsions (MASC 2)	54.00 [46.50–61.00]	53.00 [43.00–61.00]	0.009
Physical symptoms (MASC 2)	59.00 [47.00–70.00]	56.00 [50.00–67.00]	0.009
Panic (MASC 2)	55.00 [42.00–69.00]	58.00 [53.00–64.00]	0.080
Tense/restless (MASC 2)	59.00 [46.00–66.00]	52.00 [42.00–67.00]	0.086
Harm avoidance (MASC 2)	54.00 [46.00–60.00]	54.00 [43.00–60.00]	0.047
Anxiety probability score (MASC 2)	1.00 [0.00–3.00]	1.00 [0.00–2.00]	0.003
Inconsistency index (MASC 2)	6.00 [5.00–7.00]	7.00 [6.00–8.00]	0.284
BUT (mean)	2.23 [1.65–3.38]	1.32 [0.69–2.39]	0.292
Drive for thinness (EDI-3)	14.00 [3.00–27.00]	13.00 [9.00–21.00]	0.019
Bulimia (EDI-3)	0.00 [0.00–4.00]	1.00 [0.00–6.00]	0.162
Body dissatisfaction (EDI-3)	20.00 [13.00–25.00]	19.00 [13.00–27.00]	0.015
Low self-esteem (EDI-3)	8.00 [4.00–13.00	8.00 [2.00–12.00]	0.080
Personal alienation (EDI-3)	7.00 [4.00–17.00]	7.00 [5.00–13.00]	0.077
Interpersonal insecurity (EDI-3)	12.00 [9.00–13.00]	10.00 [8.00–12.00]	0.254
Interpersonal alienation (EDI-3)	6.00 [4.00–12.00]	6.00 [3.00–10.00]	0.146
Interoceptive deficits (EDI-3)	12.00 [4.00–23.00]	10.00 [6.00–19.00]	0.069
Emotional dysregulation (EDI-3)	8.00 [2.00–12.00]	8.00 [4.00–14.00]	0.015
Perfectionism (EDI-3)	6.00 [4.00–10.00]	10.00 [5.00–14.00]	0.184
Ascetism (EDI-3)	5.00 [3.00–14.00]	6.00 [4.00–9.00]	0.012
Maturity fears (EDI-3)	12.00 [9.00–25.00]	12.00 [8–.00–17.00]	0.134
Eating disorder risk (EDI-3)	39.00 [22.00–54.00]	38.00 [23.00–44.00]	0.050
Ineffectiveness (EDI-3)	15.00 [7.00–31.00]	14.00 [8.00–24.00]	0.092
Interpersonal problems (EDI-3)	19.00 [14.00–24.00]	14.00 [13.00–21.00]	0.165
Affective problems (EDI-3)	18.00 [13.00–35.00]	20.00 [12.00–27.00]	0.042
Overcontrol (EDI-3)	13.00 [8.00–24.00]	16.00 [11.00–23.00]	0.050
General psychological maladjustment (EDI-3)	84.00 [53.00–145.00]	77.00 [63.00–104.00]	<0.001
Problem solving (FAD)	1.83 [1.67–2.00]	2.00 [2.00–2.25]^*^	0.421
Communication (FAD)	2.33 [1.95–2.56]	2.61 [2.17–2.84]	0.260
Roles (FAD)	1.91 [1.73–2.14]	2.09 [2.00–2.14]	0.296
Affective responsiveness (FAD)	2.17 [1.92–2.60]	2.25 [2.00–2.59]	0.035
Affective involvement (FAD)	1.79 [1.57–1.93]	2.00 [1.72–2.29]	0.274
Behavior control (FAD)	2.00 [1.84–2.28]	2.22 [1.95–2.28]	0.177
General functioning (FAD)	1.88 [1.50–2.17]	2.04 [1.79–2.42]	0.312

*Data are presented as median and interquartile range in square brackets [IQR] for continuous variables. BUT, Body Uneasiness Test; CDI 2, Children depression inventory 2; EDI-3, Eating Disorder Inventory-3; FAD, Family Assessment Device; MASC 2, Multidimensional Anxiety Scale for Children 2; IP, inpatient care; HLCT, high-level care treatment; YSR, Youth Self-Report. Mann–Whitney test was used to compare continuous variables between the two groups. Statistical significance for p < 0.05*.

* *p < 0.05. Effect size (r) was calculated for the Mann–Whitney test. Small, medium, and large effects for r < 0.3, 0.3 ≤ r < 0.5, and r ≥ 0.5, respectively*.

The patients' clinical pathways have been checked at T1 with the MROAS. Differences, with medium to large effect size, were found for the following scales: eating difficulties (Mann–Whitney *U* = 26.500, *p* < 0.001, *r* = 0.730), mental state (Mann–Whitney *U* = 75.000, *p* = 0.028, *r* = 0.437), insight (Mann–Whitney *U* = 38.500, *p* < 0.001, *r* = 0.635), intimate relationships (Mann–Whitney *U* = 58.000, *p* = 0.004, *r* = 0.523), social contacts (Mann–Whitney *U* = 60.000, *p* = 0.005, *r* = 0.509), and occupation (Mann–Whitney *U* = 36.500, *p* < 0.001, *r* = 0.687). The Chi-square test performed on the MROAS groups confirmed an improvement in the HLCT group compared to the IP one, showing a large effect size (*p* = 0.003, Cramer's *V* = 0.592). Table 4 summarizes the scores for each scale and the MROAS groups in both IP and HLCT patients.

**Table 4:** MROAS at T1 in IP and HLCT patients.

	**IP**	**HLCT**	**Effect size (*r* or Cramer's *V*)**
MROAS scales		
Eating difficulties	3.00 [3.00–4.00]	2.00 [2.00–2.5]^***^	0.730
Menstrual state	4^a^	4.00 [4.00–4.00]	0.263
Mental state	4.00 [4.00–4.00]	3.00 [2.00–4.00]^*^	0.437
Insight	3.00 [3.00–4.00]	2.00 [1.00–2.00]^***^	0.635
Intimate relationships	4.00 [3.00–4.00]	2.50 [2.00–3.00]^**^	0.523
Family relationships	3.00 [3.00–3.00]	3.00 [2.00–3.00]	0.230
Social contacts	3.00 [3.00–4.00]	2.50 [2.00–3.00]^**^	0.509
Occupation	3.00 [3.00–3.00]	2.00 [2.00–2.00]^***^	0.687
Additive behaviors	1^a^	1^a^	0.000
MROAS groups		
Good	0 (0.0)	3 (17.6)^**^	0.592
Intermediate	2 (11.8)	8 (47.1)	
Poor	15 (88.2)	5 (29.4)	

*Data are presented as number and percentage in parentheses (%) for categorical variables, or median and interquartile range in square brackets [IQR] for continuous variables. HLCT, high-level care treatment; MROAS, Morgan–Russel Outcome Assessment Scale; IP, inpatient care. Mann–Whitney and Chi-square tests were used to compare continuous and categorical variables between groups, respectively*.

a *Constant values. Statistical significance for p < 0.05*.

* *p < 0.05*,

** *p < 0.01*,

*** *p < 0.001. Effect size (r or Cramer's V) was calculated for the Mann–Whitney test or the Chi-square test, respectively. Small, medium, and large effects for r < 0.3, 0.3 ≤ r < 0.5 and r ≥ 0.5, respectively*.

### Hospital Costs

The IP group showed a median cost of € 2,267.00 [2,267.00–2,340.00] per patient, while in the HLCT group, it was € 3,240.00 per patient [2,106.00–4,374.00]. The Mann–Whitney test was performed in order to compare costs between the two treatments. No difference was found (Mann–Whitney *U* = 163.000, *p* = 0.540, *r* = 0.111).

## Discussion

This retrospective pilot study aimed to describe the first available data on an Italian partial hospitalization program—the HLCT treatment—in order to evaluate the impact on patients' clinical status (in terms of weight recovery and mental state), average hospitalization days, and hospital costs between the HLCT and IP treatments for adolescents with AN.

A first result indicates that patients from both groups share the same clinical parameters (weight, pBMI, and HR), illness characteristics (length of illness, rate of weight loss, time of weight loss, and prior treatments) and psychological problems: showing no difference in degree of disease severity at T0.

The only two differences, with a medium effect size, noted between the two groups was the higher reporting of negative self-esteem by patients in the IP group while there was a higher perception of difficulty in regard to parental problem-solving skills in the HLCT group. These results could be read within the different care contexts, where the more restrictive measures adopted in our IP treatment compared to the HLCT program may trigger greater feelings of guilt, ineffectiveness, and passivity in the patient with respect to their treatment path. On the contrary, in the HLCT program, the patient continues to eat meals, even at home, with all the difficulties in place, and this possibly could contribute to the child feeling greater parental difficulty in managing the food symptom alone.

While both groups started with the same initial clinical conditions, the HLCT group had a lower number of treatment days, less frequent use of fluid therapy and enteral nutrition needs and a faster attainment of conditions required for discharge than did the IP group. In our programs, discharge from both the inpatient and HLCT programs occurs when the medical parameters are stabilized, there is a constant increase in weight gain, and patients have begun to show a greater adherence to the dietary plan. Subsequently, they are referred to treatments with a lower weekly frequency, such as the MIT (12).

Patients in the HLCT group seems to present a greater increase in pBMI, with a large effect size, compared to the IP group, also showing a greater effect size in terms of weight recovery. When evaluating the patients at the end of their treatment programs using the MROAS, results with large effect size emerged for the HLCT group when compared to the IP group in terms of progress in several categories such as social contact, occupation (school), intimate relationships, insight, mental states, and eating difficulties. This may indicate that a faster constant weight gain, in terms of change in pBMI points, may also correspond to a greater openness to the psychological reflections underlying the eating disorder and to a less rigidity regarding nutrition. It seems useful to point out that these results could also derive from a selection bias, where, in the absence of randomization, HLCT patients were more compliant with meals, more motivated and not in a risk condition. However, the current literature (31) underlines that letting the patient maintain normal social and work activities favors patient compliance and accelerates the treatment process, not only in terms of weight recovery but also with respect to psychological characteristics, such as the ability to think about oneself and their illness. Obviously, AN treatment does not end with the discharge from the IP or HLCT programs, but continues and changes in intensity and type according to the treatment path. Hence, it becomes important to understand how to reduce hospitalizations or partial hospitalization times, in order to allow patients and families to access, as soon as possible, a treatment more focused on relational and intrapsychic difficulties rather than nutritional aspects.

Finally, we have calculated hospital costs for the IP and HLCT treatments. Results show no difference regarding the cost to the hospital between the two therapeutic approaches, with the median cost for the IP program being € 2,267.00 and 3,240.00 for the HLCT program. This result is not in line with our expectations or with the international published literature that underlines a lower cost for partial hospitalizations with respect to inpatient treatment programs (8). We hypothesize that the reason for this result is related to the method used for our calculations: the DRG system used in Italy considers the average cost per diagnosis, and not the direct costs incurred for the individual interventions and procedures implemented for each patient the introduction of the HLCT program represents a meeting point between inpatient and outpatient treatment, combining the merits of both treatments. HLCT shows greater results both in terms of weight recovery and in terms of psychic and relational functioning for equivalent patient groups with equal costs to traditional treatment programs. It is clear that in cases of serious risk to life, ordinary hospitalization is inevitable, which however could be limited to the rebalancing of medical parameters, favoring a subsequent transfer into a care setting that allows, on the one hand, multidisciplinary and continuous assistance and, on the other hand, maintenance of social and relational activities. A further benefit would also be reducing the time of hospitalization and therefore a greater ability to accept new patients.

Our study, the first on the Italian adolescent population, is in line with the recent literature and confirms the need to deepen the investigation into the benefits of partial hospitalization vs. full hospitalization through a randomized clinical trial.

The work presented has several limitations. The study design was observational, retrospective, and non-randomized. Therefore, all patients admitted to the two different treatments were included in the analysis, according to the including/excluding criteria applied a posteriori. Despite the observational design, no difference in body weight, BMI, length of illness, prior treatments, weight loss, the time in which it occurred, and psychological characteristics at the admission was detected between the two groups. For this reason, a comparison between them at T1 (discharge from each treatment) was performed, sensing that there was a minor risk of bias. Moreover, the small sample size may have limited the ability to detect differences between IP and HLCT, and the short treatment period did not allow for the re-administration of the same battery of tests at T1, so the MROAS scale was used at T1, in order to assess the biopsychosocial outcomes. It is possible that most severe patients were more represented in the IP group, partially explaining the differences in outcomes. Moreover, psychological treatment in the IP program is less intensive compared to the HLCT program because, even if patients have daily clinical monitoring, psychotherapy sessions occur only once a week instead of twice as in the HLCT program. From a clinical point of view, this difference may represent a minor curbing of the patients' anxieties related to greater adherence to dietary indications, with a possible consequence of a slower process of treatment and development of compliance. Further prospective and randomized studies are needed in order to better investigate the different treatment programs for severe AN in adolescence.

## Data Availability Statement

The raw data supporting the conclusions of this article will be made available by the authors, without undue reservation.

## Ethics Statement

The studies involving human participants were reviewed and approved by Ethical Committees of the Bambino Gesù Children Hospital (2264_OPBG_2020). Written informed consent to participate in this study was provided by the participants' legal guardian/next of kin.

## Author Contributions

VZ and SV planned the study. MC, GC, MM, CC, and IC collected the data. SG performed the economic evaluation of the costs. GC and AT performed the statistical analysis. MC, MCC, AC, and IC analyzed the literature. VZ, MC, and GC wrote the manuscript. All authors contributed to the article and approved the submitted version.

## Conflict of Interest

The authors declare that the research was conducted in the absence of any commercial or financial relationships that could be construed as a potential conflict of interest.
