# Corrigendum: Improvements on Clinical Status of Adolescents With Anorexia Nervosa in Inpatient and Day Hospital Treatment: A Retrospective Pilot Study

**DOI:** 10.3389/fpsyt.2021.748046

**Published:** 2021-08-17

**Authors:** Valeria Zanna, Giulia Cinelli, Michela Criscuolo, Anna Maria Caramadre, Maria Chiara Castiglioni, Ilenia Chianello, Maria Rosaria Marchili, Chiara Casamento Tumeo, Stefano Guolo, Alberto Eugenio Tozzi, Stefano Vicari

**Affiliations:** ^1^Anorexia Nervosa and Eating Disorder Unit, Child Neuropsychiatry, Department of Neuroscience, Bambino Gesù Children's Hospital, Istituto di ricovero e cura a carattere scientifico (IRCCS), Rome, Italy; ^2^Predictive and Preventive Medicine Research Unit, Bambino Gesù Children's Hospital, Istituto di ricovero e cura a carattere scientifico (IRCCS), Rome, Italy; ^3^General Pediatrics Unit, Department of Emergency, Acceptance and General Pediatrics, Bambino Gesù Children's Hospital, Istituto di ricovero e cura a carattere scientifico (IRCCS), Rome, Italy; ^4^Sanitary Direction, Bambino Gesù Children's Hospital, Istituto di ricovero e cura a carattere scientifico (IRCCS), Rome, Italy; ^5^Child and Adolescent Psychiatric Unit, Department of Neuroscience, Bambino Gesù Children's Hospital, Istituto di ricovero e cura a carattere scientifico (IRCCS), Rome, Italy

**Keywords:** daily treatment, inpatient care, hospital care, partial hospitalization, anorexia nervosa, adolescence

In the published article, an author name was incorrectly spelled as Annamaria Caramadre. The correct spelling is Anna Maria Caramadre.

In the published article, there was an error in the affiliation of authors Giulia Cinelli and Alberto Eugenio Tozzi. Instead of affiliation 1, it should be affiliation 2. Conversely, the affiliation of the author Ilenia Chianello should be affiliation 1 instead of affiliation 2.

The authors apologize for these errors and state that this does not change the scientific conclusions of the article in any way. The original article has been updated.

## Publisher's Note

All claims expressed in this article are solely those of the authors and do not necessarily represent those of their affiliated organizations, or those of the publisher, the editors and the reviewers. Any product that may be evaluated in this article, or claim that may be made by its manufacturer, is not guaranteed or endorsed by the publisher.

